# A New Instrument (Distinguisher) for Radioscopy

**Published:** 1912-10

**Authors:** 


A NEW INSTRUMENT (DISTINGUISHER) FOR RADIOSCOPY. Guido Holknecht, *Archives d'Electricite Medicale,* Oct., 1911. Manual pressure has been employed to bring out more distinctly shadows of certain organs in abdominal radioscopy but the hand is subjected to rays passing through the patient. To avoid this danger, the author has invented a crank-shaped instrument ending in a spoon-like pressor, the recurved crank coming out in front of the screen with a handle. The hand, holding this handle or knob is protected by the lead glass of the radioscopic screen from the X-rays.

